# *trnL* outperforms *rbcL* as a DNA metabarcoding marker when compared with the observed plant component of the diet of wild white-faced capuchins (*Cebus capucinus*, Primates)

**DOI:** 10.1371/journal.pone.0199556

**Published:** 2018-06-26

**Authors:** Elizabeth K. Mallott, Paul A. Garber, Ripan S. Malhi

**Affiliations:** 1 Department of Anthropology, Northwestern University, Evanston, IL, United States of America; 2 Department of Anthropology, University of Illinois at Urbana-Champaign, Urbana, IL, United States of America; 3 Carl R Woese Institute for Genomic Biology, University of Illinois at Urbana-Champaign, Urbana, IL, United States of America; University of Hyogo, JAPAN

## Abstract

DNA metabarcoding is a powerful tool for assessing the diets of wild animals, but there is no clear consensus on which proposed plant barcoding marker is most suitable for dietary analysis. This study compares two DNA plant barcoding markers that are commonly used for dietary analyses from degraded DNA, *rbcL* and *trnL*, to detailed dietary observations of wild white-faced capuchins (*Cebus capucinus*). Observational dietary data and fecal samples (n = 170) were collected for one year from a group of individually recognizable monkeys at La Suerte Biological Field Station, Costa Rica. DNA was extracted and portions of the *rbcL* and *trnL* chloroplast were amplified and sequenced on the Illumina MiSeq platform. Sequences were analyzed using *obitools*. Of the two barcoding markers tested, *trnL* yielded greater numbers of sequences with equal sequencing effort, higher resolution taxonomic identifications (albeit with a larger reference database), and identified a greater number of families also found in the observed diet. There was no relationship between observed capuchin feeding behavior and dietary composition based on either sequence occurrence or relative abundance of sequences using *rbcL* as a marker. However, dietary composition based on the relative abundance of *trnL* sequences was significantly positively associated with the observed percentage of feeding and foraging time capuchins’ spent on each plant species. Additionally, in 35% of cases, the relative abundance of *trnL* sequences assigned to particular plant families in fecal samples was highly positively correlated with time spent consuming plants from those same families. Our results indicate that *trnL* is a more robust DNA metabarcoding marker for plant dietary analysis and may potentially be used to quantitatively assess differences in diet within or between species.

## Introduction

DNA metabarcoding is quickly becoming a powerful tool in ecology for understanding trophic interactions and non-invasively assessing the diets of wild animals, particularly with the increasing availability of high-throughput sequencing technologies [1,2]. However, DNA barcoding marker genes must be chosen carefully to provide meaningful results [3]. The choice of marker depends on the biology of the study species, the questions being asked, the quality of the DNA sample, and completeness of reference databases [2]. Target regions must be variable enough to discriminate between closely related animal and plant species, yet flanking regions should be conserved enough to be less variable within a single species. Additionally, primer sets should readily amplify a range of species within a large taxonomic group. Finally, the target region must be long enough to yield the desired taxonomic resolution. Even though there is a consensus for some taxonomic groups as to which standardized DNA region should be used for metabarcoding, including dietary analysis (e.g., *COI* for invertebrates) [4,5], multiple barcoding target regions have been proposed for other taxonomic groups, such as plants [1,3,6,7].

An additional important consideration for dietary analysis using DNA metabarcoding is the ability of a primer to amplify often degraded and low copy number DNA from fecal samples. Mini-barcodes that target regions of DNA that are generally 150bp or smaller allow for the amplification of degraded DNA [8,9]. While these smaller barcoding regions are preferable for degraded samples [3,8,10,11], smaller barcoding regions result in less specific taxonomic identifications [12]. Additionally, in plants, using single locus barcodes can lead to less reliable taxonomic identifications due to high rates of hybridization, introgression, and polyploidy in land plants [13].

Two single-locus regions, *trnL* and *rbcL*, have been proposed for dietary analysis using DNA metabarcoding in herbivores and omnivores [1,3,14]. In particular, the trnL-g and trnL-h primer set used to amplify the P6 loop of *trnL* (UAA) [1,15–23] and the rbcLZ1 and rbcL19b mini-barcoding primers used to amplify a small region of *rbcL* [14,23–25] have been widely used. A few studies have directly compared the performance of *rbcL* and *trnL* as barcoding regions [23–26]. Results indicated that *trnL* had higher amplification success and a larger sequence divergence range in a study of wetland plants [25], while *rbcL* had a more complete publicly available reference database [26]. Both regions generally were only able to provide taxonomic resolution at the level of genus or family [9]. In addition, rates of sequence divergence, and, therefore, taxonomic resolution of mini-barcodes in these two regions may differ depending on the plant family or families of interest in a particular study [25].

Relatively less is known, however, about how dietary results from each of these barcoding regions compare to other methods of assessing diet, particularly how molecular results compare to fine-scale dietary observations over long time periods. In one study where sika deer (*Cervus nippon*) were fed a known diet, DNA metabarcoding using *trnL* was able to reliably detect all of the foods present in the diet when a local reference database was used [20]. However, taxonomic identifications were limited to the genus or family level when using a global database in the same study [20]. Similarly, in a study of red-shanked douc langurs (*Pygathrix nemaeus*) fed a known diet, DNA metabarcoding using *trnL* was only able to identify genera known to be in the diet in 31.3–37.5% of cases using a global reference database [27]. Studies comparing microhistological analysis of fecal samples or macroscopic analysis of stomach contents with DNA metabarcoding, using either *trnL* or *rbcL* as markers, found varying amounts of overlap between taxa identified by the two methods [15,18,23,28–30]. Many of these studies also showed that DNA metabarcoding provided much greater taxonomic resolution than microhistology [15,18,28,29]. Few studies have compared results from one of the DNA metabarcoding markers with methods of direct observation of diet in a wild mammal. In a study of woodland caribou (*Rangifer tarandus caribou*), Newmaster *et al*. (2013) found that results from DNA metabarcoding using *trnL* and a global reference database and results from animal-borne video recording were highly correlated. Similarly, Hargrave (2015) found that DNA metabarcoding using *trnL* and a global reference database supplemented with local plant species sequences identified all but one taxon of plants that wild sable antelope (*Hippotragus niger*) were observed to have consumed. In a study of black and white colobus monkeys (*Colobus guereza*), Bradley *et al*. (2007) found that DNA metabarcoding using *rbcL* and a global reference database identified plants known to be part of an individual’s diet during detailed behavioral observations taken during the previous two days. However, the study failed to detect some plants that an individual was observed eating and identified plants that were not recorded as part of an individual’s diet but may have been consumed [24]. None of these studies have compare the precision of both *rbcL* and *trnL* with direct detailed dietary observations collected over more than six months.

To fill this gap, here we evaluate the precision and taxonomic resolution of DNA metabarcoding-based dietary analysis using both *rbcL* and *trnL*. We compare molecularly-determined diet with detailed individual-level dietary observations of a group of wild white-faced capuchins (*Cebus capucinus*) over a year-long period. We also assess whether the relative abundance of *rbcL* or *trnL* reads is correlated with the percentage of time spent feeding or foraging on food items.

## Materials and methods

### Study site and population

**Fig 1:**
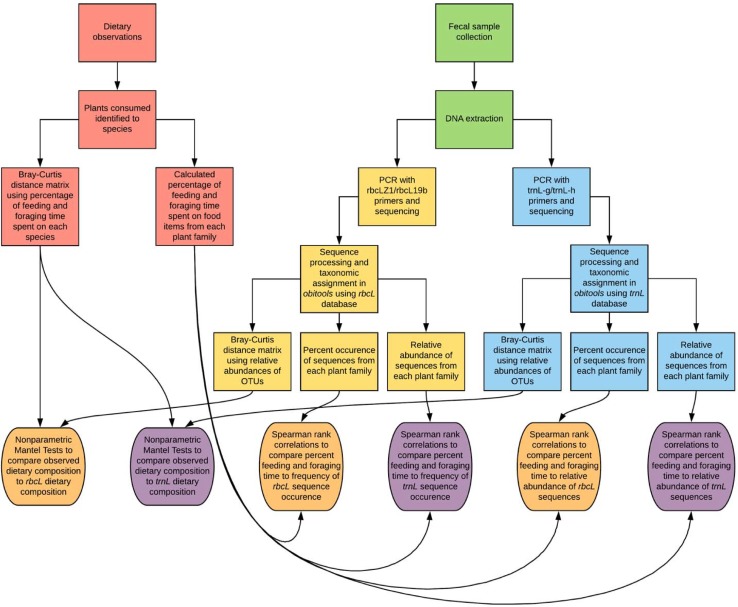
Summary of sample collection and analysis. Flowchart showing sample collection, sample processing for each type of sample, and statistical analyses using the resulting data from each sample type.

The study was conducted from January 2013 through January 2014 at La Suerte Biological Field Station in northeastern Costa Rica (10.445N, 83.784W). La Suerte Biological Field Station includes 170 ha of advanced secondary wet tropical forest and 130 ha of early secondary growth and regenerating pasture [31]. Annual rainfall during the study period was 3116 mm. A group of individually recognizable white-faced capuchins (*Cebus capucinus*) containing 21–22 individuals during the study period was observed (a detailed description of the study site and population is found in Mallott *et al*. 2017) [32]. An overview of sample collection and analysis is found in Fig 1 (Fig 1).

### Observational data collection

Dietary data were collected during one-hour instantaneous focal animal samples (two-minute interval) of individually recognizable adult males, adult females, and juveniles. Data were collected between 0500 and 1600 hours, and 841 hours of dietary data were collected over 237 days. Activity budget (feeding–actively consuming food items; foraging–manipulating or searching for food items; traveling–movement between or within the crowns of trees which was not for the immediate purpose of obtaining food or engaging in social activity; resting–periods of inactivity; social–engaged in any affiliative or agonistic interaction with at least one other individual) was recorded at each two-minute interval. If an individual was feeding or foraging, diet (ripe fruit, unripe fruit, seeds, flowers, leaves, invertebrates, vertebrates, other) was recorded. Plant food items were identified to species, when possible, and identifications were aided by reference materials in the library at La Suerte Biological Field Station and the knowledge of station staff. Overall, 80% of the plant species consumed by the capuchins were identified to the species level and 86% of plant feeding and foraging records were a known species.

Fecal samples were collected from all individuals throughout the observation study period (n = 170) in sterile tubes, fixed in 90% ethanol, and stored at -20C. The mean number of fecal samples collected per month was 13.1±5.3 (range = 4–21). To minimize environmental contamination, portions of fecal material in direct contact with soil or leaf litter were not collected. CITES export permits were obtained from Sistema Nacional de Areas de Conservación in Costa Rica, and import permits for the United States were obtained from the CDC. Data collection protocols were approved by the University of Illinois IACUC, La Suerte Biological Field Station, the Ministerio de Ambiente, Energía, y Telecomunicaciones in Costa Rica, and the Comisión Nacional para la Gestión de la Biodiversidad in Costa Rica.

### Molecular data collection

A QIAamp DNA Stool Mini Kit was used to extract DNA from 170 fecal samples, following the provided protocol for “Isolation of DNA from Stool for Human DNA Analysis” (using the modifications outlined in Mallott *et al*. 2015) [33]. A 157bp segment of the *rbcL* gene was amplified using the rbcLZ1 and rbcL19b primers [14,24], and a 22-143bp segment of the *trnL* (UAA) intron was amplified using the trnL-g and trnL-h primers [3]. Negative extraction and PCR controls were used to minimize the risk of contamination. Samples were individually indexed, using the same barcode for both *rbcL* and *trnL* amplicons for each sample, and the amplicons were amplified on a Fluidigm Access Array system at the Roy J. Carver Biotechnology Center at UIUC (for reaction conditions and detailed methods see Mallott *et al*. 2017). Amplicons were sequenced on the Illumina MiSeq v2 platform with paired end 2x300 bp reads. In *obitools*, raw sequences were paired with an alignment score threshold of 20, assigned to samples, de-duplicated, low-count sequences and PCR errors were removed, and the remaining sequences were assigned to taxa using the *ecotag* script and reference databases for both *rbcL* and *trnL* [34]. References databases for *rbcL* and *trnL* were created using *ecoPCR* [35] from release 131 of the European Nucleotide Archive. The *rbcL* database contained 1589 unique plant sequences which include 92 orders, 284 families, 881 genera, and 1054 species. The *trnL* database contained 13941 unique plant sequences which include 75 orders, 365 families, 4028 genera, and 8656 species. Tab-delimited sequence read count tables were created in *obitools*, and the resulting files were collapsed at the operational taxonomic unit, genus, and family levels. Relative abundances were then calculated in the *vegan* package [36] of R (r-project.org). Details of the bioinformatics analyses can be found in the supporting information (S1 File). Raw sequence data is available in the Sequence Read Archive (http://www.ncbi.nlm.nih.gov/sra), BioProject ID PRJNA299397, and the *ecotag* output for both the *rbcL* and *trnL* sequences can be found in the supporting information (S1 and S2 Tables).

### Statistical methods

Nonparamentric Mantel tests were used to compare Bray-Curtis dissimilarity matrices of monthly individual observed and molecular diets. Relative abundances of sequences were averaged across the month for individuals for whom we had multiple molecular samples in the same month. To assess relationships between diet analysis methods at the group-level, instead of individual-level, spearman rank correlations were used to test for a positive association between the percentage of the capuchin group’s feeding and foraging time spent consuming plants from a specific family, and the frequency of occurrence of sequences assigned to that family (percentage of fecal samples containing sequences assigned to a family) and average relative abundance of sequences assigned to that same family within a given month for both *rbcL* and *trnL* sequencing results.

## Results

### Observed diet

During the study period, 47.8% of observed white-faced capuchin feeding and foraging time was spent on fruit, 49.8% on invertebrates, 1.2% on flowers, 0.5% on leaves, 0.01% on vertebrates, and 0.6% on other plant foods. *Psidium guajava* (Myrtaceae) accounted for 21.2% of feeding and foraging time on fruit items. *Hampea appendiculata* (Malvaceae) (9.5%), *Dipteryx panamensis* (Fabaceae) (8.5%), *Inga spectabilis* (Fabaceae) (6.8%), *Urera baccifera* (Urticaceae) (6.5%), and *Inga thibaudiana* (Fabaceae) (6.3%) also accounted for a substantial percentage of white-faced capuchin feeding and foraging time (Table 1). Fifty-nine species of fruit from 27 plant families were consumed during the study period (for detailed dietary results, see S3 Table).

**Table 1:** Observed fruit diet based on the percentage of feeding and foraging time spent on each fruit species.

Species	Family	Percentage of diet	Average monthly percentage	Months eaten
Psidium guajava	Myrtaceae	21.2%	19.0% (±10.8%)	All
Hampea appendiculata	Malvaceae	9.5%	13.7% (±22.9%)	Jan, Feb, Nov, Dec
Dipteryx panamensis	Fabaceae	8.5%	6.9% (±10.8%)	Jan—Jun
Inga spectabilis	Fabaceae	6.8%	4.5% (±8.6%)	Feb—May, Oct
Urera baccifera	Urticaceae	6.5%	4.1% (±12.6%)	Oct—Dec
Inga thibaudiana	Fabaceae	6.3%	4.6% (±9.6%)	Jan, Mar—May, Jul—Dec
Ficus colubrinae	Moraceae	4.1%	4.0% (±3.8%)	Feb, Mar, May—Aug, Oct—Dec
Ficus trigonata	Moraceae	3.7%	2.5% (±6.8%)	Apr, May, Oct, Nov
Conostegia xalapensis	Melastomataceae	3.4%	2.8% (±3.8%)	Jan, Apr—Jun, Aug, Sep, Nov, Dec
Sapium grandulosum	Euphorbiaceae	2.8%	1.4% (±3.0%)	Jul, Aug, Oct, Nov
Ficus schippii	Moraceae	2.2%	2.6% (±5.6%)	May, Jun, Nov
Inga marginata	Fabaceae	2.1%	2.0% (±3.2%)	Jan, Feb, Jun, Aug, Dec
Ficus tonduzii	Moraceae	1.8%	1.2% (±3.1%)	May, Oct
Nephelium lappaceum	Sapindaceae	1.7%	1.7% (±3.0%)	Jun—Oct
Cestrum megalophylum	Solanaceae	1.7%	1.1% (±4.1%)	Dec
Dendropanax arboreus	Araliaceae	1.6%	1.6% (±5.4%)	Sep, Oct
Piper spp.	Piperaceae	1.3%	1.0% (±1.1%)	Feb, Apr, Nov, Dec
Miconia affinis	Melastomataceae	1.2%	1.1% (±2.3%)	Apr—Jun, Aug, Sep
Casearia arborea	Salicaceae	1.1%	1.1% (±2.3%)	Jun, Oct, Nov
Miconia elata	Melastomataceae	1.1%	0.5% (±0.0%)	Apr, May, Aug, Nov

### Molecular dietary analysis results

Sequencing of *rbcL* amplicons yielded 1706 unique sequences, after dereplicating and denoising the sequence dataset. Thirty-six families, 39 genera, and 36 species were identified using *rbcL* (Table 2 and S4 Table). Sequences assigned to Solanaceae were the most frequently occurring *rbcL* sequences in the fecal samples, occurring in 99.4% of samples. Sequences assigned to Melastomataceae (97.1%), Araceae (88.2%), Moraceae (78.8%), Myrtaceae (66.5%), Urticaceae (47.7%), Fabaceae (41.8%), and Convolvulaceae (40.0%) also were among the most frequently occurring *rbcL* sequences (Table 2). These families also accounted for the highest relative abundances of *rbcL* sequences, with Solanaceae accounting for 62.9% of sequences assigned to family, Melastomataceae for 17.7%, Araceae for 8.0%, Moraceae for 4.0%, Myrtaceae for 2.6%, and Urticaceae for 1.5%.

**Table 2:** Percentage of observed feeding and foraging records assigned to each family of fruit and frequency of occurrence and relative abundance of *rbcL* and *trnL* sequences over the course of the entire study period.

Family	Percentage of observed fruit feeding and foraging records	Frequency of occurrence of *rbcL* sequences	Average relative abundance of *rbcL* sequences	Total relative abundance of *rbcL* sequences	Frequency of occurrence of *trnL* sequences	Average relative abundance of *trnL* sequences	Total relative abundance of *trnL* sequences
Acanthaceae	-	-	-	-	11.76%	0.02%	0.01%
Annonaceae	-	-	-	-	74.71%	2.16%	1.93%
Apiaceae	-	1.18%	0.01%	0.00%	-	-	-
Apocynaceae	0.20%	-	-	-	-	-	-
Araceae	-	88.24%	11.26%	7.99%	-	-	-
Araliaceae	1.58%	-	-	-	-	-	-
Arecaceae	0.78%	-	-	-	33.53%	0.62%	0.76%
Asteraceae	-	-	-	-	0.59%	0.00%	0.00%
Bignoniaceae	0.32%	12.35%	1.08%	0.31%	31.76%	0.22%	0.26%
Boraginaceae	0.09%	1.76%	0.01%	0.00%	-	-	-
Brassicaceae	-	0.59%	0.00%	0.00%	-	-	-
Bromeliaceae	-	-	-	-	60.00%	1.64%	2.76%
Burseraceae	-	-	-	-	18.82%	0.17%	0.13%
Calyceraceae	-	19.41%	0.96%	0.85%	-	-	-
Canellaceae	-	-	-	-	3.53%	0.00%	0.00%
Capparaceae	-	-	-	-	1.76%	0.01%	0.01%
Caprifoliaceae	-	1.18%	0.06%	0.01%	-	-	-
Caricaceae	-	-	-	-	28.24%	0.66%	2.33%
Chenopodiaceae	-	1.76%	0.00%	0.00%	-	-	-
Chrysobalanaceae	-	-	-	-	2.94%	0.01%	0.00%
Clusiaceae	0.11%	17.65%	2.09%	0.44%	24.12%	0.63%	0.01%
Convolvulaceae	0.06%	40.00%	0.24%	0.11%	18.24%	0.01%	0.00%
Cordiaceae	-	-	-	-	10.00%	0.02%	0.00%
Costaceae	-	0.59%	0.04%	0.00%	7.06%	0.13%	0.00%
Cucurbitaceae	-	8.82%	0.01%	0.00%	-	-	-
Cyclanthaceae	-	2.94%	0.01%	0.02%	-	-	-
Euphorbiaceae	2.90%	4.12%	0.22%	0.11%	83.53%	2.66%	2.03%
Fabaceae	25.09%	41.76%	4.37%	0.40%	99.41%	5.65%	2.13%
Gesneriaceae	-	2.35%	0.07%	0.02%	2.35%	0.01%	0.01%
Heliconiaceae	0.34%	-	-	-	-	-	-
Hypoxidaceae	-	-	-	-	2.35%	0.00%	0.00%
Iteaceae	-	4.71%	0.01%	0.00%	-	-	-
Lamiaceae	0.43%	17.65%	0.90%	0.47%	25.29%	0.58%	0.09%
Lauraceae	0.06%	2.35%	0.01%	0.01%	24.71%	0.12%	0.18%
Lecythidaceae	-	21.18%	0.41%	0.00%	27.65%	0.05%	0.00%
Malpighiaceae	0.06%	-	-	-	-	-	-
Malvaceae	10.05%	2.35%	0.18%	0.01%	89.41%	5.52%	7.08%
Marantaceae	-	-	-	-	2.35%	0.06%	0.00%
Melastomataceae	6.17%	97.06%	15.11%	17.73%	80.00%	2.17%	1.73%
Moraceae	16.19%	78.82%	10.14%	4.01%	60.00%	3.42%	0.72%
Musaceae	0.17%	-	-	-	98.82%	3.48%	2.55%
Myristicaceae	0.17%	-	-	-	0.59%	0.01%	0.00%
Myrtaceae	21.27%	66.47%	9.64%	2.64%	100.00%	40.69%	49.90%
Onagraceae	-	2.94%	0.00%	0.00%	-	-	-
Orchidaceae	-	2.35%	0.04%	0.00%	-	-	-
Orobanchaceae	-	0.59%	0.00%	0.00%	0.59%	0.00%	0.00%
Papaveraceae	-	7.06%	0.03%	0.00%	-	-	-
Passifloraceae	-	-	-	-	24.71%	0.08%	0.09%
Piperaceae	1.29%	-	-	-	90.00%	4.33%	3.31%
Platanaceae	-	-	-	-	23.53%	0.16%	0.02%
Poaceae	-	-	-	-	7.06%	0.09%	0.02%
Primulaceae	-	-	-	-	1.18%	0.00%	0.00%
Rhamnaceae	-	-	-	-	24.71%	1.28%	0.10%
Rosaceae	-	2.35%	0.00%	0.00%	-	-	-
Rubiaceae	1.12%	11.18%	0.67%	0.11%	47.06%	1.29%	1.26%
Salicaceae	1.15%	-	-	-	-	-	-
Sapindaceae	1.72%	-	-	-	58.24%	0.74%	0.68%
Sapotaceae	0.26%	-	-	-	22.94%	0.14%	0.28%
Simaroubaceae	0.17%	-	-	-	2.35%	0.20%	0.02%
Solanaceae	1.69%	99.41%	36.61%	62.90%	97.65%	15.24%	16.39%
Strelitziaceae	-	0.59%	0.00%	0.00%	-	-	-
Trochodendraceae	-	2.35%	0.12%	0.00%	-	-	-
Typhaceae	-	-	-	-	4.12%	0.00%	0.00%
Ulmaceae	-	5.29%	0.00%	0.00%	-	-	-
Urticaceae	6.55%	47.65%	4.97%	1.53%	85.88%	5.66%	3.06%
Velloziaceae	-	-	-	-	0.59%	0.00%	0.00%
Zingiberaceae	-	16.47%	0.75%	0.33%	20.59%	0.02%	0.12%

Average relative abundances are calculated across all fecal samples collected during the study period.

Sequencing of *trnL* amplicons yielded 4551 unique sequences. Forty-five families, 68 genera, and 69 species were identified using *trnL* (Table 2 and S5 Table). Sequences assigned to Myrtaceae were the most frequently occurring *trnL* sequences in the fecal samples, occurring in 100% of samples. Sequences assigned to Fabaceae (99.4%), Musaceae (98.8%), Solanaceae (97.7%), Piperaceae (90.0%), Malvaceae (89.4%), Urticaceae (85.9%), Euphorbiaceae (83.5%), and Melastomataceae (80.0%) were among the most frequently occurring *trnL* sequences (Table 2). Many of these families also had high relative abundances of *trnL* sequences, with Myrtaceae accounting for 50% of sequences assigned to family, Solanaceae for 16.4%, Malvaceae for 7.1%, Piperaceae for 3.3%, and Urticaceae for 3.1%.

Overall, sequencing of *trnL* amplicons resulted in 311% more sequences and 267% more unique sequences, likely partially due to the shorter length of the *trnL* amplicon. A higher percentage of *trnL* sequences were identified to both order and family, a higher percentage of observed families also were identified with *trnL*, and the *trnL* method resulted in a greater number of identified families, genera, and species when compared with *rbcL* (Table 3). This result is not surprising, however, given the larger size of the *trnL* reference database used for this study. A greater percentage of *rbcL* sequences were identified at the levels of genus and species (Table 3), which is surprising as the *trnL* database contains four times as many genera and eight times as many species as the *rbcL* database. Of the 67 total families identified in this study, 14 were detected with all three methods and 19 were detected with both *rbcL* and *trnL* (Table 3). While the uneven distribution of fecal samples across months likely contributed to the fact that neither *rbcL* or *trnL* detected all species of food present in the observational diet, 92.5% of plant families found in the observation diet were also found using the DNA metabarcoding methodology (Table 3).

**Table 3:** Comparison of *rbcL* and *trnL* sequencing performance, resolution, and accuracy.

	*rbcL*	*trnL*
**Sequences**	2,541,027	7,910,288
**Unique sequences**	1706	4551
**Percentage identified to order**	53.8%	89.7%
**Percentage identified to family**	47.2%	79.2%
**Percentage identified to genus**	9.1%	8.2%
**Percentage identified to species**	7.7%	2.0%
**Identified families also in observed diet**	14	21
**Total number of families identified**	67	
**Families identified with all three methods**	14	
**Families identified with both *rbcL* and *trnL***	19	
**Families only identified with observational data**	5	
**Families only identified with *rbcL***	16	
**Families only identified with *trnL***	20	

### Comparison of observed and molecularly determined diet

Non-parametric Mantel tests showed a significant positive correlation between the observed dietary composition, based on feeding and foraging time in a given month, and dietary composition for each month determined with *trnL* (ρ = 0.1025, p = 0.045). These results indicate that individuals with more similar monthly diets assessed with DNA metabarcoding using *trnL* had more similar monthly diets based on feeding and foraging observations. However, the same was not true for *rbcL*. There was no relationship between observed monthly plant dietary composition and monthly dietary composition determined with *rbcL* (ρ = 0.0653, p = 0.145).

**Fig 2:**
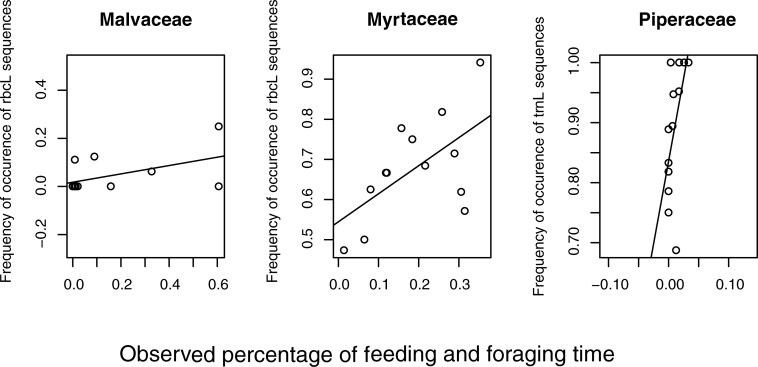
Relationship between feeding and foraging time and frequency of occurrence of *rbcL* and *trnL* sequences. Correlations between observed percentage of group feeding and foraging time spent on each plant family in a given month and the frequency of occurrence of *rbcL* or *trnL* sequences in the same month.

There was no relationship between the frequency of occurrence of either *rbcL* or *trnL* sequences assigned to a family in fecal samples in a given month and the percentage of feeding and foraging time the group was observed to be eating food items from that same family in that month (S6 Table). The exceptions to this were the significant positive correlations between the frequency of occurrence of *rbcL* sequences assigned to Myrtaceae and percentage of feeding and foraging time spent consuming Myrtaceae (ρ = 0.4869, p = 0.046), and the frequency of occurrence of *trnL* sequences assigned to Piperaceae and percentage of feeding and foraging time spent consuming Piperaceae (ρ = 0.6763, p = 0.006) (Fig 2).

**Fig 3:**
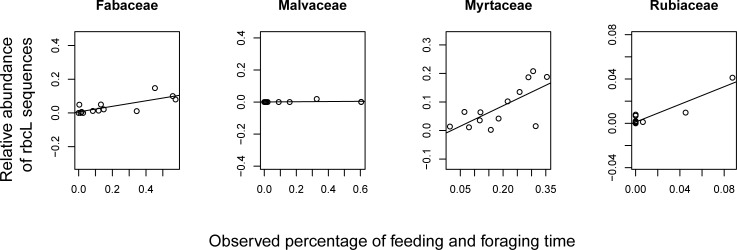
Relationship between feeding and foraging time and relative abundance of *rbcL* sequences. Correlations between observed percentage of group feeding and foraging time spent on each plant family in a given month and the average relative abundance of *rbcL* sequences for the same month.

**Fig 4:**
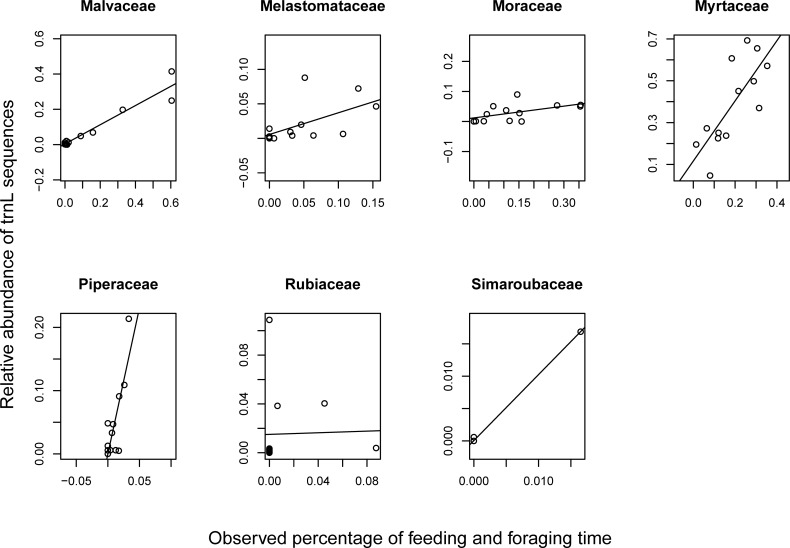
Relationship between feeding and foraging time and relative abundance of *trnL* sequences. Correlations between observed percentage of group feeding and foraging time spent on each plant family in a given month and the average relative abundance of *trnL* sequences for the same month.

There were several plant families for which the average relative abundance of *rbcL* or *trnL* sequences were significantly positively correlated with the percentage of feeding and foraging time the group spent consuming that same family in a given month (S6 Table). The average relative abundance of *rbcL* sequences assigned to Fabaceae, Malvaceae, Myrtaceae, and Rubiaceae were significantly positively correlated with the percentage of monthly feeding and foraging time spent exploiting those same families (ρ = 0.7473, p = 0.002; ρ = 0.5077, p = 0.038; ρ = 0.5840, p = 0.016; ρ = 0.5840, p = 0.018) (Fig 3 and S6 Table). The average relative abundance of *trnL* sequences assigned to Malvaceae, Melastomataceae, Moraceae, Myrtaceae, Piperaceae, Rubiaceae, and Simaroubaceae were significantly positively correlated with the percentage of feeding and foraging time spent on those same families (ρ = 0.8118, p<0.001; ρ = 0.6658, p = 0.007; ρ = 0.5604, p = 0.025; ρ = 0.7418, p = 0.003; ρ = 0.6104, p = 0.013; ρ = 0.5722, p = 0.021; ρ = 0.5654, p = 0.022) (Fig 4 and S6 Table). Of note, several of these families (Fabaceae, Malvaceae, Melastomataceae, Moraceae, and Piperaceae) were among the most frequently observed families consumed during the 12-month study period by this group of white-faced capuchins. There was a significant positive relationship between percentage of monthly feeding and foraging time spent consuming species from a particular family and the average relative abundance of sequences assigned to that family in four cases for *rbcL* and in seven cases for *trnL*.

## Discussion

This study compared detailed observational dietary data with molecular dietary analysis using DNA metabarcoding of both *rbcL* and *trnL* chloroplast genes in a group of individually recognizable, wild white-faced capuchin monkeys. The results showed that using most metrics, *trnL* outperformed *rbcL*. Using *trnL* as a barcoding marker resulted in more sequences, more unique sequences, a greater percentage of sequences that could be identified to order or family, and results that were more comparable to those obtained from observational data. *rbcL* did have a greater percentage of sequences that could be identified to genus and species (9.1% vs. 8.2% and 7.7% vs. 2.0%), but neither marker had high rates of successful taxonomic assignments at these levels. This is not unexpected in light of previous work showing that neither *rbcL* or *trnL* have particularly high rates of species-level discrimination, particularly when compared with barcoding markers used in other organisms [1,3,6,7,13,37,38]. While these results may indicate that *trnL* has greater taxonomic resolution than *rbcL*, the difference in the number and identity of plant species present in both reference databases makes the difference in resolution difficult to interpret. Genus- and species-level identifications would likely be improved with the use of a local reference plant DNA database for both markers. However, the lack of a good reference database is often the case in studies using DNA metabarcoding, particularly when the diet of the study species is not well characterized. Even without a local reference database, it appears that the correlation between results obtained from *trnL* sequences and the observed diet make *trnL* a good candidate for a barcoding marker that can be used to make inferences concerning broader questions about dietary differences between and within species living in the same habitats.

Some of the discrepancies between the three different identification methods are not surprising. The four families of plants identified only during behavioral observations are infrequently eaten plants–each making up less than 2% of the total white-faced capuchin diet (Table 2). Thus, it is likely that we would not have a fecal sample collected from an individual who consumed those foods items within the past eight hours. Similarly, many of the families of plants that were only identified using the *trnL* method–Annonaceae, Burseraceae, Caricaceae, Cordiaceae (Boraginaceae), Passifloraceae, and Rhamnaceae–are plants that are known to be part of the white-faced capuchin diet, either at this site or other sites in Costa Rica, but are infrequently consumed [39–41]. Other plant families identified by only the *rbcL* method (Araceae, Cyclanthaceae, and Ulmaceae) or the *trnL* method (Acanthaceae, Bromeliaceae, and Marantaceae) include plant species that are consumed by white-faced capuchins [39,40,42], but the flower or leaves are consumed, so they were not recorded as part of the fruit diet in this study. Both the *rbcL* method and the *trnL* method identified plant families that have not yet been recorded as part of the white-faced capuchin diet, but are present in this area of Costa Rica, and so may be rarely consumed by this group of white-faced capuchins. For the *rbcL* method, these include Brassicaceae, Caprifoliaceae, Curcubitatceae (either from crop raiding chayote trees from neighboring farms or consuming small undomesticated variants), Onagraceae, Orchidaceae, Papaveraceae, Rosaceae, and Strelitziaceae (while this family does not have any species native to Costa Rica, there are several ornamental varieties on the field station property). For the *trnL* method, these potentially rarely consumed families of plants include Asteraceae, Canellaceae, Capparaceae, Poaceae, Primulaceae, and Typhaceae. Both molecular methods also assigned sequences to plant families that are not found in Costa Rica (*rbcL*: Calyceraceae, Iteaceae, and Trochodendraceae; *trnL*: Platanaceae and Velloziaceae), and likely represent taxonomy assignment errors due to limitations of the reference databases.

For the majority (66.7%) of plant foods eaten by white-faced capuchins, our results indicate that frequency of occurrence of both *rbcL* and *trnL* sequences assigned to a given family in fecal samples was not correlated with the percentage of feeding and foraging time spent consuming fruits assigned to that same family. However, for plant foods such as Myrtaceae and Malvaceae, the relative abundances of *rbcL* and *trnL* sequences assigned to a give family were predictive of how much feeding and foraging time the white-faced capuchin group spent consuming fruits from that same family. This was particularly true of the relative abundance of *trnL* sequences and foods that account for at least 5% of white-faced capuchin monkey fruit consumption. Though these results suggest a predictive relationship between the relative abundance of *trnL* sequences and the relative time spent consuming fruits, the use of DNA metabarcoding to obtain quantitative dietary results is an area of active research. Whereas some studies have found no relationship between the relative abundance of *trnL* sequences and observational or microhistology data [20,30], other studies have had greater success. Srivathsan *et al*. (2015) found that the relative abundance of *trnL* sequences assigned to given genus was highly correlated with the amount of foods consumed from that genus by captive red-shanked douc langurs. In a study of large African herbivores, raw read abundance of *trnL* sequences assigned to grasses were tightly correlated with δ^13^C values and estimates of relative C_4_-plant consumption from stable isotope data [10].

Though the results of our study suggest that *trnL* is a more robust DNA metabarcoding marker for dietary analysis and that relative abundances of *trnL* sequences may be able to be used to assess relative changes or differences in diet, the results should be interpreted cautiously. While percentage of feeding and foraging time is frequently used in primatological studies to track diet, it is not necessarily an accurate approximation of the amount of plant foods consumed [43], as individuals may consume different foods at different rates and individuals of different age, sex, or dominance status might consume different foods and in different proportions. Controlled feeding studies where the amount of a food consumed and gut passage rates are known are imperative to more precisely determine whether or not relative abundances of *trnL* can accurately be used to quantify the relative contributions of plants foods to animal diets or to compare changes in diet within or between species. Additionally, the extraction kit used likely contains DNA from plants in the Solanaceae family [1], resulting in contamination of our sequencing results. This may be responsible for the fact that Solanaceae DNA were present in a high proportion of fecal samples despite there only being observations of plants in the Solanaceae family being consumed in one month (December). Future studies examining the impact of variation in sample storage methods, extraction methods, PCR protocols, and primer biases would be beneficial.

Comparing *trnLg/h* to other minibarcodes also would be beneficial. Several other barcodes have been proposed for plants, including *trnH-psbA* and *ITS*, and a multi-locus tiered approach using *rbcL* and *matK* has been selected as the barcode of choice by the CBOL Plant Working Group [6,7,9,13,44,45]. Several studies have used minibarcodes from these genes, or combinations of two DNA barcoding regions, to identify the plant component of animal diets [10,22,24,25,46,47], whereas other studies have used minibarcodes in different subsections of the *rbcL* gene [12,29]. Therefore, even though the *trnL* method outperformed the *rbcL* method in the context of the current study, other DNA barcoding approaches for determining the plants present in animal diets may be more accurate.

The *trnL* barcoding marker yielded results more similar to those from detailed behavioral observations when compared with the *rbcL* barcoding marker for DNA metabarcoding analyses of omnivorous and herbivorous diets. These results indicate that *trnL* is a more universal primer set with less amplification biases than rbcL, at least when amplifying plant DNA from fecal samples. Our data suggests that relative abundances of *trnL* sequences can be used to approximate relative time spent consuming a plant food, particularly for frequently consumed foods. However, additional studies are necessary to determine whether DNA metabarcoding using *trnL* can be used to accurately predict relative rates of consumption of plant foods.

## Supporting information

S1 File Detailed methods for the bioinformatic analyses. (DOCX)

S1 Table *ecotag* output for *rbcL* sequences with taxonomic annotations. (TXT)

S2 Table *ecotag* output for *trnL* sequences with taxonomic annotations. (TXT)

S3 Table Percentage of observed feeding and foraging time spent consuming plant species in each month. (XLSX)

S4 Table Relative abundance of *rbcL* sequences assigned specific taxa for the entire study period. (XLSX)

S5 Table Relative abundance of *trnL* sequences assigned specific taxa for the entire study period. (XLSX)

S6 Table Correlations between observed percentage of feeding and foraging time on plant families and both frequency of occurrence and relative abundances of *rbcL* and *trnL* sequences. (XLSX)
