# Supplementary material for: trnL outperforms rbcL as a DNA metabarcoding marker when compared with the observed plant component of the diet of wild white-faced capuchins (Cebus capucinus, Primates)
Source: PLoS One. 2018 Jun 26;13(6):e0199556. doi: 10.1371/journal.pone.0199556 (PMC6019260; doi:10.1371/journal.pone.0199556)
Supplement: S1 File — (DOCX) [file pone.0199556.s001.docx]

**Bioinformatic analysis**

Reference databases for both *rbcL* and *trnL* were created using *ecoPCR* (Ficetola *et al.* 2010), release 131 of the European Nucleotide Archive, and the current NCBI taxonomy (as of 06/06/2017). The following commands were used:

obiconvert --embl -t ./taxo --ecopcrDB-output=embl_6617 ./embl/*.dat

ecoPCR -d ./embl_6617 -e 3 -l 50 -L 500 GGGCAATCCTGAGCCAA CCATTGAGTCTCTGCACCTATC > trnl.ecopcr

obigrep -d embl_6617 --require-rank=species --require-rank=genus --require-rank=family trnl.ecopcr > trnl_clean.fasta

obiuniq -d embl_6617 trnl_clean.fasta > trnl_clean_uniq.fasta

obigrep -d embl_6617 --require-rank=family trnl_clean_uniq.fasta > trnl_clean_uniq_clean.fasta

obiannotate --uniq_id trnl_clean_uniq_clean.fasta > db_trnl.fasta

ecoPCR -d ./embl_6617 -e 3 -l 50 -L 500 ATGTCACCACAAACAGAGACTAAAGCAAGT CTTCTTCAGGTGGAACTCCAG > rbcl.ecopcr

obigrep -d embl_6617 --require-rank=species --require-rank=genus --require-rank=family rbcl.ecopcr > rbcl_clean.fasta

obiuniq -d embl_6617 rbcl_clean.fasta > rbcl_clean_uniq.fasta

obigrep -d embl_6617 --require-rank=family rbcl_clean_uniq.fasta > rbcl_clean_uniq_clean.fasta

obiannotate --uniq_id rbcl_clean_uniq_clean.fasta > db_rbcl.fasta

The resulting *rbcL* database had 1589 unique plant sequences and the resulting *trnL* database had 1589 unique plant sequences.

Raw sequence reads were paired, cleaned, de-deduplicated, assigned to taxonomy, and a tab-delimited taxonomy file was created in *obitools* (Riaz *et al.* 2011; Shehzad *et al.* 2012). The analysis followed the tutorial found at <http://metabarcoding.org/obitools/doc/wolves.html>, with a few modifications. For the *illuminapairedend* step, a minimum score of 20 was used. As barcodes had been removed in the original sequence processing at UIUC and reads were already demultiplexed, *ngsfilter* was run with -:- in the mapping file in place of the barcode tag sequence. The following commands were then used for each sequencing file (one file existed per sample):

obigrep -a ‘error:Cannot assign sequence to a sample’ SampleID.unidentified.fastq > SampleID.good.fastq

obiannotate -S ‘sample:SampleID’ SampleID.good.fastq > SampleID.id.fastq

The following command was used to concatenate the above files and dereplicate the sequences:

obiuniq -m sample *.id.fastq > all_uniq.fasta

For the *obigrep* step to remove low count and short sequences, a count threshold of greater than or equal to 10 sequences was used, and no minimum sequence length was used.

**References**

Ficetola GF, Coissac E, Zundel S *et al.* (2010) An in silico approach for the evaluation of DNA barcodes. *BMC Genomics*, **11**, 434.

Riaz T, Shehzad W, Viari A *et al.* (2011) ecoPrimers: inference of new DNA barcode markers from whole genome sequence analysis. *Nucleic Acids Research*, **39**, e145.

Shehzad W, Riaz T, Nawaz MA *et al.* (2012) Carnivore diet analysis based on next-generation sequencing : application to the leopard cat (Prionailurus bengalensis) in Pakistan. *Molecular Ecology*, **21**, 1951–1965.
